# Supplementary material for: Transcription–replication conflicts underlie sensitivity to PARP inhibitors
Source: Nature. 2024 Mar 20;628(8007):433–41. doi: 10.1038/s41586-024-07217-2 (PMC11006605; doi:10.1038/s41586-024-07217-2)
Supplement: Supplementary file 1 — Supplementary Figs. 1 and 2 containing the uncropped blots and gating strategy. [file 41586_2024_7217_MOESM1_ESM.pdf]

nature portfolio

https://doi.org/10.1038/s41586-024-07217-2

Supplementary information

Transcription–replication conflicts underlie sensitivity to PARP inhibitors

In the format provided by the authors and unedited

Supplementary Fig. 1a

Uncropped images of western blots for Fig. 3g.
Black rectangulars correspond the cropped images.

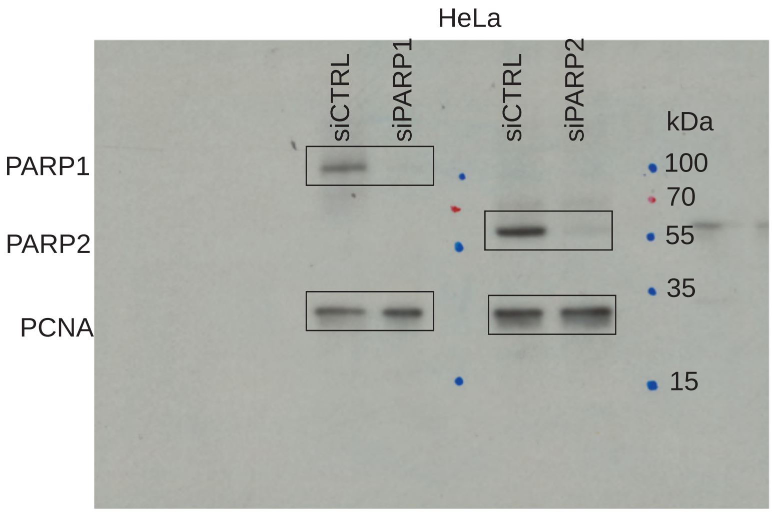

Supplementary Fig. 1b

Uncropped images of western blots for Fig. 4d.
Black rectangulars correspond the cropped images.

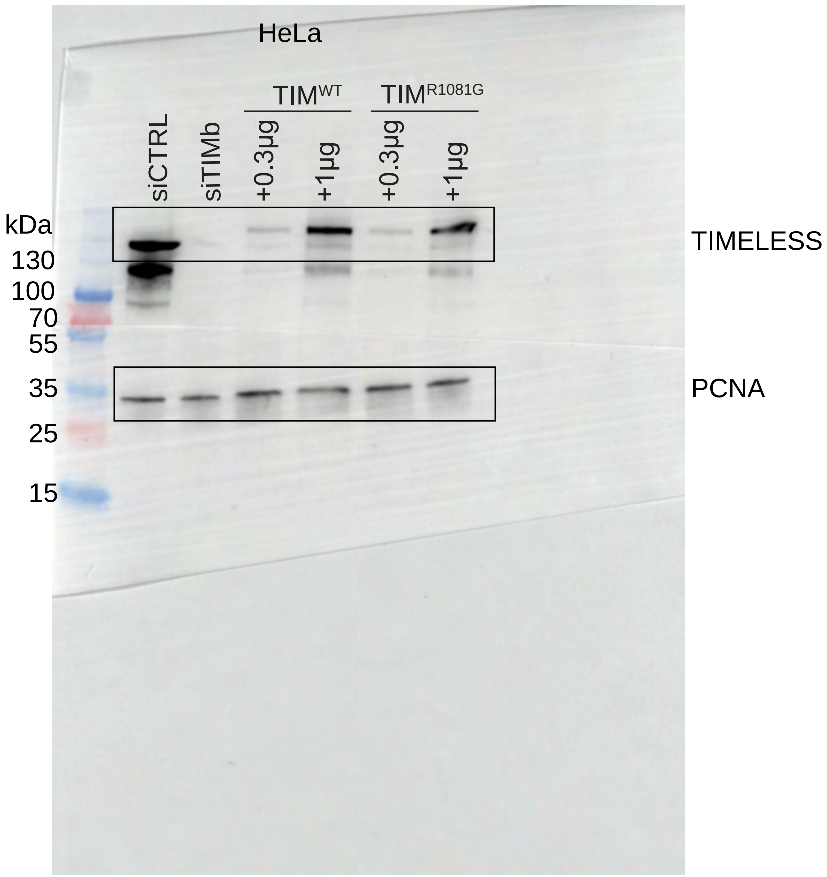

Supplementary Fig. 1c

Uncropped images of western blots for Extended Data Fig.1a.
Black rectangulars correspond the cropped images.

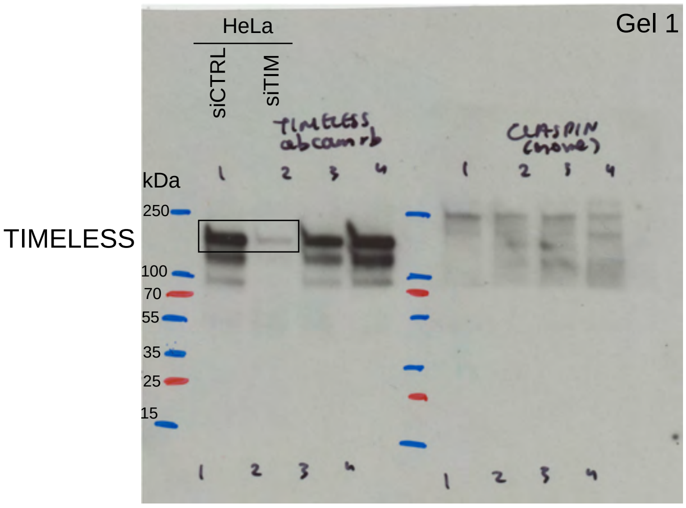

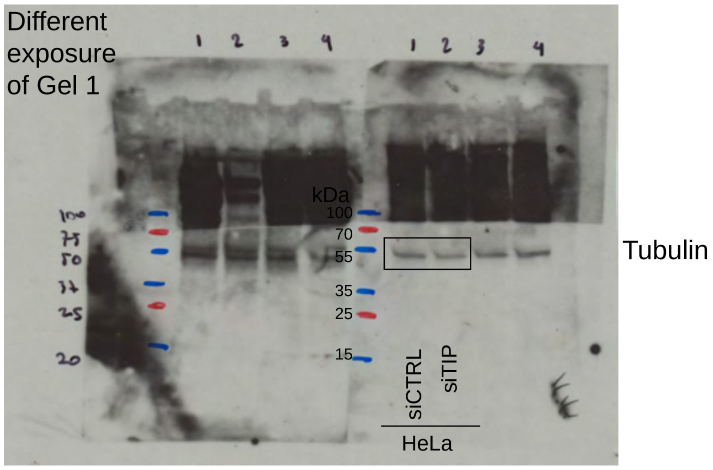

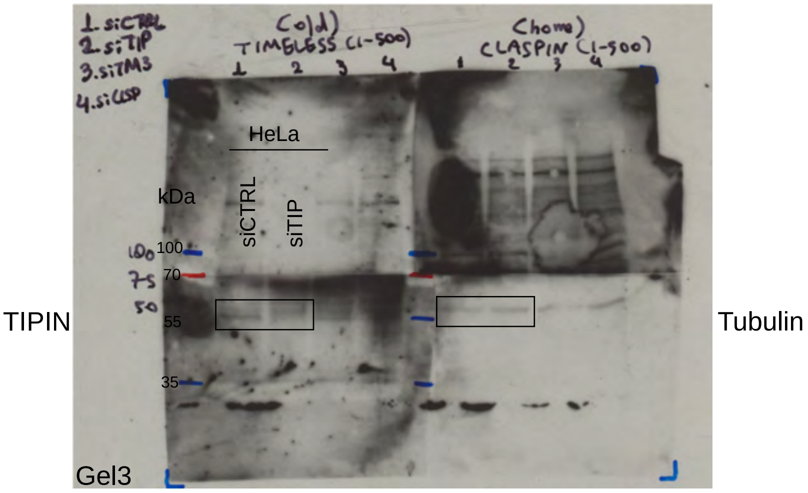

Supplementary Fig. 1d

Uncropped images of western blots for Extended Data Fig. 2b,e.
Black rectangulars correspond the cropped images.

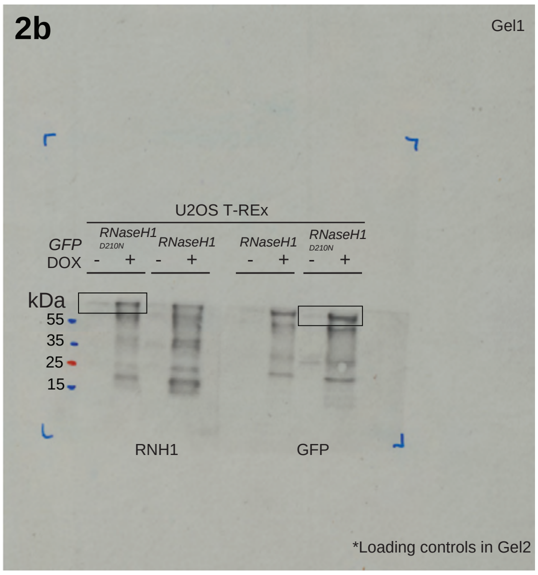

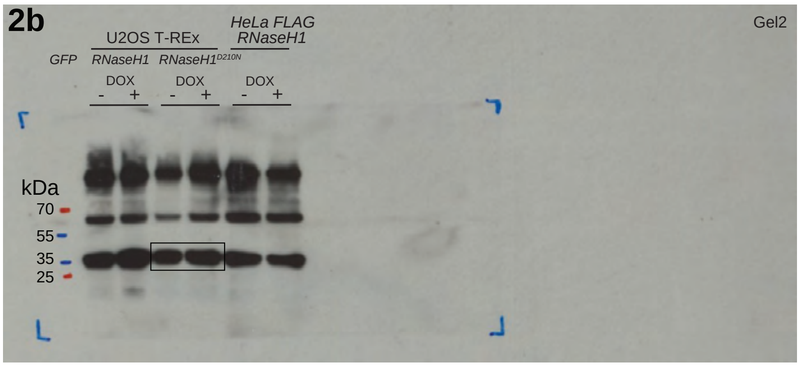

PCNA

FLAG

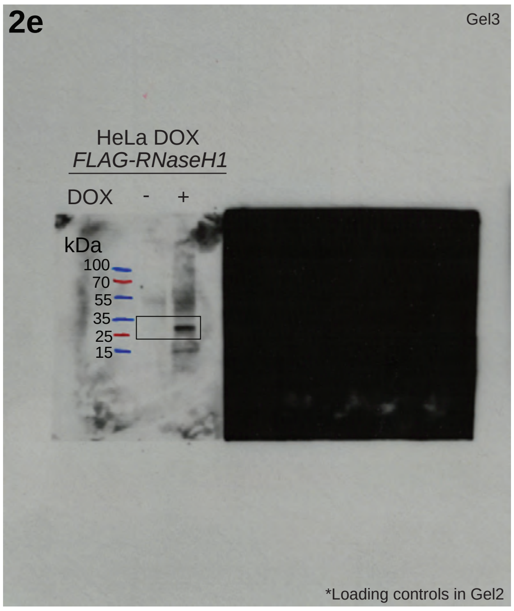

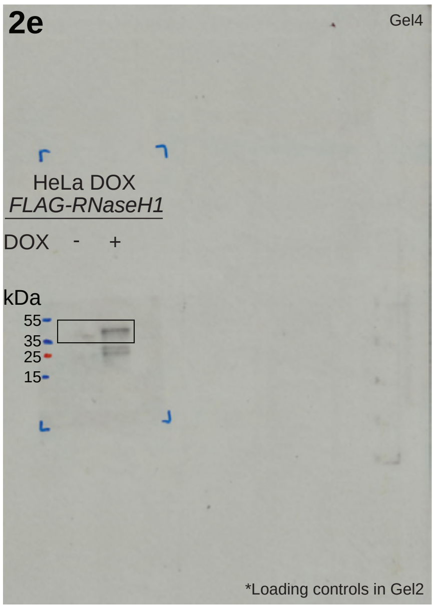

RNH1

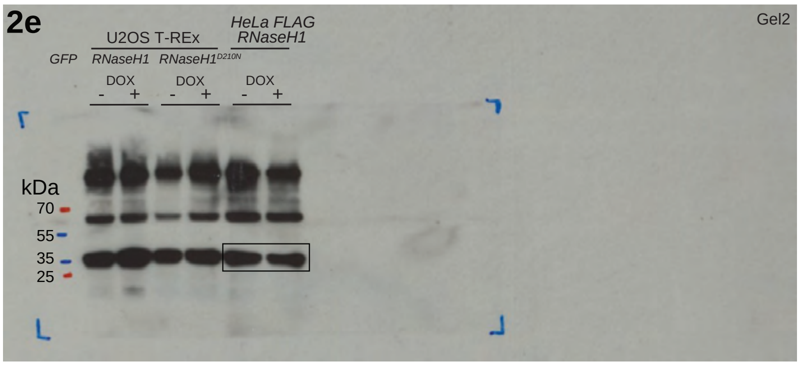

PCNA

Supplementary Fig. 1e

Uncropped images of western blots for Extended Data Fig. 5c.
Black rectangulars correspond the cropped images.

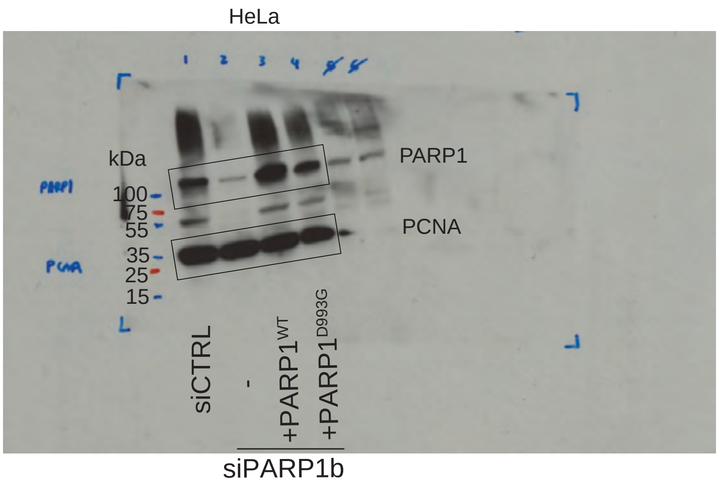

Supplementary Fig. 1f

Uncropped images of western blots for Extended Data Fig. 8b.
Black rectangulars correspond the cropped images.

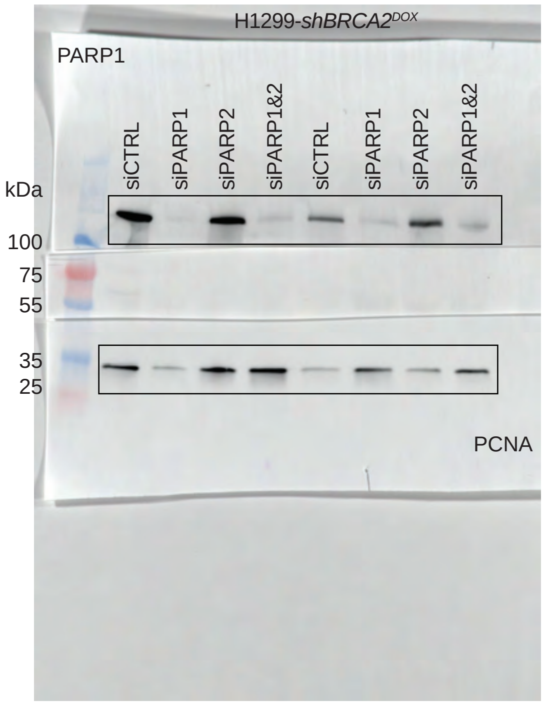

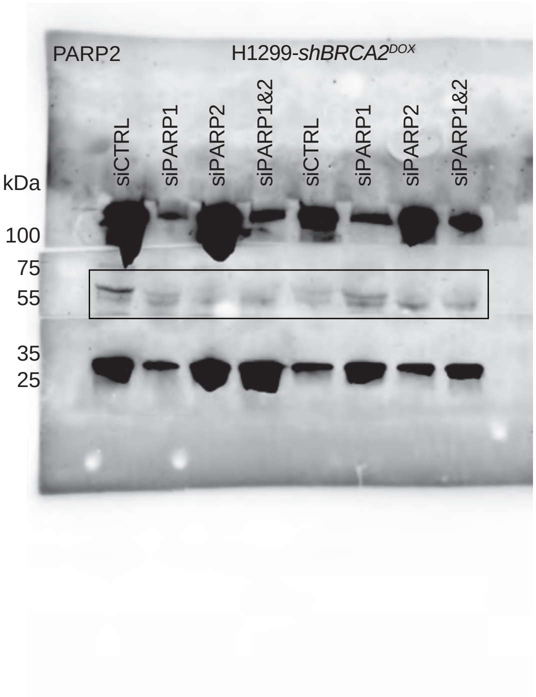

Supplementary Fig. 1g

Uncropped images of western blots for Extended Data Fig. 8b.
Black rectangulars correspond the cropped images.

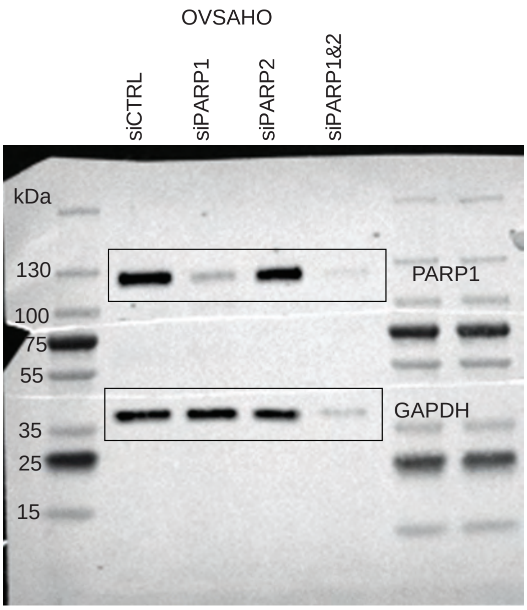

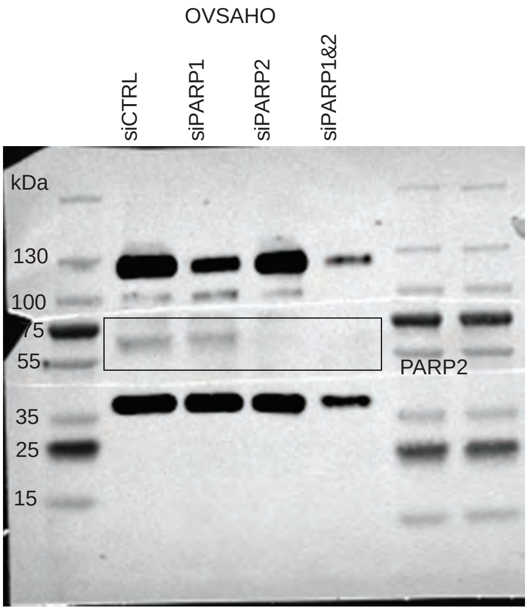

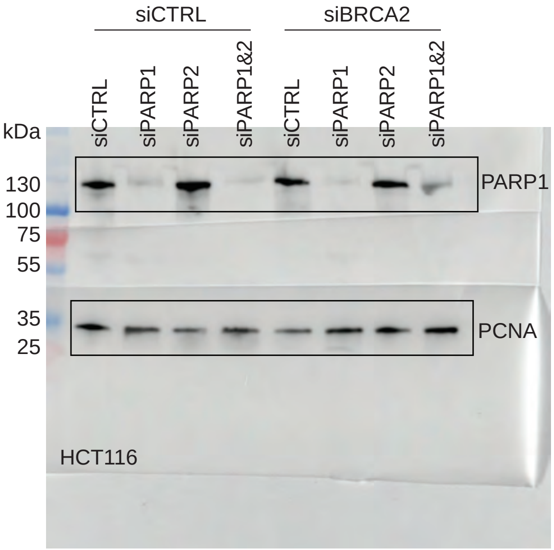

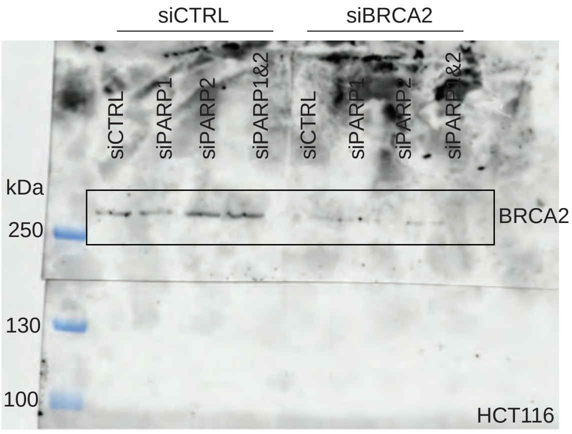

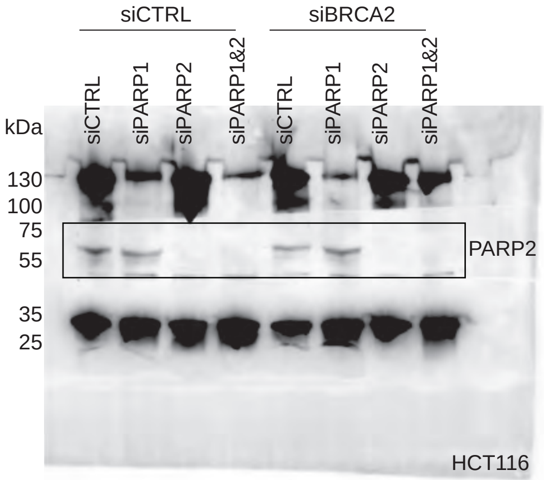

Supplementary Fig. 1h

Uncropped images of western blots for Extended Data Fig. 8b.
Black rectangulars correspond the cropped images.

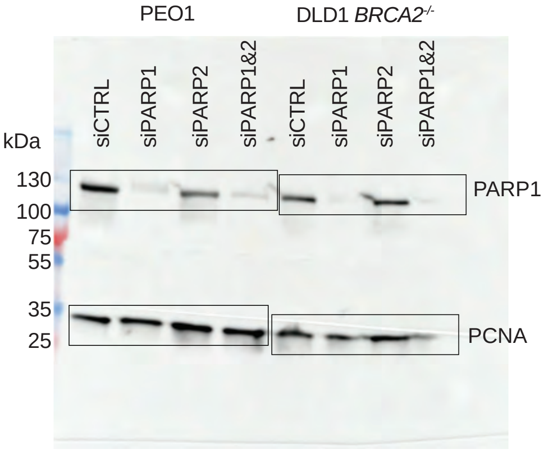

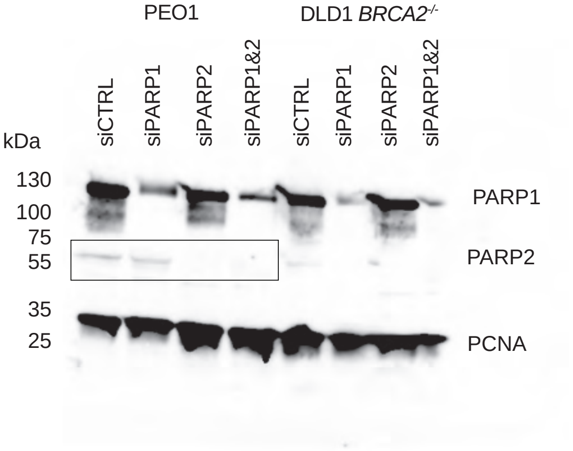

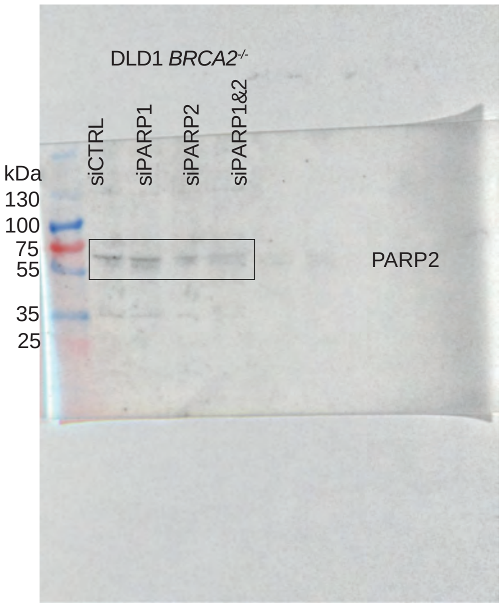

Supplementary Fig. 2

Gating strategy for flow cytometry. Intact cells were selected by a forward and side scatter gate. Then single cells were selected with a gate selecting cells with DNA content peak height and peak areas falling on a diagonal. Then gates for EdU incoorporation or above background γH2AX levels were used to select EdU positive and γH2AX positive cells.

1. Gating of intact cells by forward and side scatter

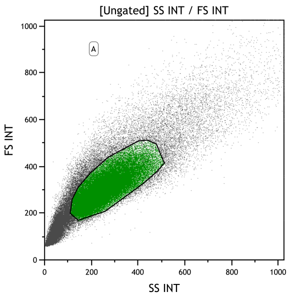

Total cells: 56.199

2. Elimination of cell doublets by plotting DNA content peak height vs peak area

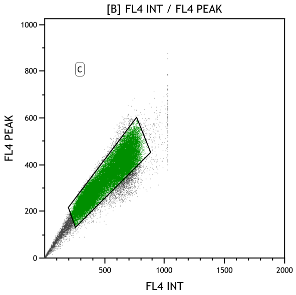

3. Gating for Edu incoorporation

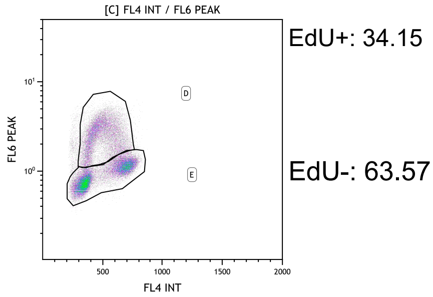

EdU+: 34.15

EdU-: 63.57

Gate	Number	%Total	%Gated
All	56,199	56.20	100.00
D	19,194	19.19	34.15
E	35,727	35.73	63.57

4. Gating for γH2AX levels

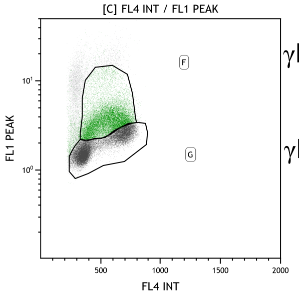

γH2AX+: 38.24

γH2AX-: 52.11

Gate	Number	%Total	%Gated
All	56,199	56.20	100.00
F	21,488	21.49	38.24
G	29,287	29.29	52.11
