# Supplementary material for: Transcription–replication conflicts underlie sensitivity to PARP inhibitors
Source: Nature. 2024 Mar 20;628(8007):433–41. doi: 10.1038/s41586-024-07217-2 (PMC11006605; doi:10.1038/s41586-024-07217-2)
Supplement: Supplementary file 2 — Reporting Summary [file 41586_2024_7217_MOESM2_ESM.pdf]

nature portfolio

Corresponding author(s): Thanos Halazonetis

Last updated by author(s): 09/02/2024

Reporting Summary

Nature Portfolio wishes to improve the reproducibility of the work that we publish. This form provides structure for consistency and transparency in reporting. For further information on Nature Portfolio policies, see our Editorial Policies and the Editorial Policy Checklist.

Statistics

For all statistical analyses, confirm that the following items are present in the figure legend, table legend, main text, or Methods section.

n/a	Confirmed	
☐	☒	The exact sample size (*n*) for each experimental group/condition, given as a discrete number and unit of measurement
☐	☒	A statement on whether measurements were taken from distinct samples or whether the same sample was measured repeatedly
☐	☒	The statistical test(s) used AND whether they are one- or two-sided<br>*Only common tests should be described solely by name; describe more complex techniques in the Methods section.*
☒	☐	A description of all covariates tested
☐	☒	A description of any assumptions or corrections, such as tests of normality and adjustment for multiple comparisons
☐	☒	A full description of the statistical parameters including central tendency (e.g. means) or other basic estimates (e.g. regression coefficient) AND variation (e.g. standard deviation) or associated estimates of uncertainty (e.g. confidence intervals)
☐	☒	For null hypothesis testing, the test statistic (e.g. *F*, *t*, *r*) with confidence intervals, effect sizes, degrees of freedom and *P* value noted<br>*Give P values as exact values whenever suitable.*
☒	☐	For Bayesian analysis, information on the choice of priors and Markov chain Monte Carlo settings
☒	☐	For hierarchical and complex designs, identification of the appropriate level for tests and full reporting of outcomes
☒	☐	Estimates of effect sizes (e.g. Cohen's *d*, Pearson's *r*), indicating how they were calculated

Our web collection on statistics for biologists contains articles on many of the points above.

Software and code

Policy information about availability of computer code

Data collection	Genomic DNA was sonicated using a Bioruptor sonicator (Diagenode). Library preparation was performed using the TruSeq ChIP Sample Prep Kit (Illumina, Cat. No. IP-202-1012). High-throughput 100-base-pair single-end sequencing was performed on an Illumina Hi-Seq 4000 sequencer. Microscopy images were acquired using a Zeiss Imager M2 AX10 with the ZEN3.4 (blue edition) software or an ImageXpress spinning disc confocal microscope (Molecular devices) with Metaepxress software. Luminescence for viability experiments was measured using a Spark 10 M microplate reader (Tecan).
Data analysis	Sequencing reads were aligned on the non-masked human genome assembly (GRCh37/hg19) using the Burrows-Wheeler Aligner software as described previously (Macheret & Halazonetis, Nature 2018; Macheret et al., Cell Research, 2020). Previously described custom Perl scripts were used to assign the aligned reads to 10 kb genomic bins. Sigma (σ) values were calculated as the normalized number of reads per bin divided by its standard deviation. The data were visualized using previously described scripts (Macheret & Halazonetis, Nature 2018). Assignment of replication timing was performed with REPLI-seq data generated previously (Macheret & Halazonetis, Nature 2018). ImajeJ version 1.8.0 and the MetaXpress Custom Module Editor was used for image analysis. Kaluza v2.1 was used for flow cytometry analysis. GraphPad Prism v9.4.1 was used for statistical analysis and graphing. Figures were assembled with Adobe Illustrator CS6.

For manuscripts utilizing custom algorithms or software that are central to the research but not yet described in published literature, software must be made available to editors and reviewers. We strongly encourage code deposition in a community repository (e.g. GitHub). See the Nature Portfolio guidelines for submitting code & software for further information.

Data

Policy information about availability of data

All manuscripts must include a data availability statement. This statement should provide the following information, where applicable:

- Accession codes, unique identifiers, or web links for publicly available datasets
- A description of any restrictions on data availability
- For clinical datasets or third party data, please ensure that the statement adheres to our policy

The fastq sequencing data and associated information described in this study have been deposited in the Sequence Read Archive (SRA) with GEO Accession Number GSE220223. The EUseq data used in this study were previously published 53. Unprocessed images of western blots and the gating strategy for the flow cytometry experiments are provided as Supplementary Information. All information supporting the conclusions are provided with the paper.

Human research participants

Policy information about studies involving human research participants and Sex and Gender in Research.

Reporting on sex and gender	N/A
Population characteristics	N/A
Recruitment	N/A
Ethics oversight	N/A

Note that full information on the approval of the study protocol must also be provided in the manuscript.

Field-specific reporting

Please select the one below that is the best fit for your research. If you are not sure, read the appropriate sections before making your selection.

☒ Life sciences ☐ Behavioural & social sciences ☐ Ecological, evolutionary & environmental sciences

For a reference copy of the document with all sections, see nature.com/documents/nr-reporting-summary-flat.pdf

Life sciences study design

All studies must disclose on these points even when the disclosure is negative.

Sample size	No statistical methods were used to determine the sample size. All experiments were performed in triplicate (independent biological triplicates) with few exceptions of experiments that were performed in duplicates (this is mentioned in the figure legends). The specific number of cells analysed for each experiment is reported in the figure legends for main and Exteded Data Figures. The experiments were performed in several different cell lines (three independent biological replicates per cell line) to determine consistency of the results across cell lines.
Data exclusions	No data were excluded.
Replication	For most of the experiments, at least three independent biological experiments were performed. For each experiment, detailed description of number of replicates, sample size and statistics is provided in figure legend. Similar parameters were evaluated by multiple methods. For example, we counted gH2AX foci by automated microscopy; and monitored total nuclear gH2AX levels by microscopy and flow cytometry, having similar results with all methods.
Randomization	Cell lines were split into different plates/wells and all control and experimental treatments were randomly assigned to the plates/wells.
Blinding	The investigator was not blinded. However, the counting of the variables was in most cases automated (eg. counting of foci, counting of immunofluorescence and flow cytometry signal intensity), so it is not possible to introduce bias by the investigator.

Reporting for specific materials, systems and methods

We require information from authors about some types of materials, experimental systems and methods used in many studies. Here, indicate whether each material, system or method listed is relevant to your study. If you are not sure if a list item applies to your research, read the appropriate section before selecting a response.

Materials & experimental systems

n/a	Involved in the study
☐	☒ Antibodies
☐	☒ Eukaryotic cell lines
☒	☐ Palaeontology and archaeology
☒	☐ Animals and other organisms
☒	☐ Clinical data
☒	☐ Dual use research of concern

Methods

n/a	Involved in the study
☐	☒ ChIP-seq
☐	☒ Flow cytometry
☒	☐ MRI-based neuroimaging

Antibodies

Antibodies used

Primary antibodies [Immunofluorescence]:
γH2AX (S139) mouse monoclonal (1:1000, clone JBW301, Millipore, Cat. No. 05-636)
RAD51 rabbit polyclonal (1:1000, Bioacademia, Cat. No. 70-002)
53BP1 rabbit polyclonal (1:1000, Novus Biologicals, Cat. No. NB100-304)
poly (ADP-ribose) mouse monoclonal (1:500, Clone 10HA, Trevigen, Cat. No. 4335-MC-100 & 1-500, Enzo Life Sciences, Cat. No. ALX-804-220-R100)
PARP1 rabbit polyclonal (1:1000, ProteinTech, Cat. No. 13371–1-AP)
PARP2 rabbit polyclonal (1:1000, Active Motif, Cat. No. 39743)

Primary antibodies [Western Blot]:
PCNA mouse monoclonal (1:1000, clone PC10, Millipore, Cat. No. MABE288)
alpha-Tubulin mouse monoclonal (1:1000, clone DM1A, Calbiochem, Cat. No. CP06)
GAPDH mouse monoclonal (1:10000, clone 6C5, Abcam, Cat. No. ab8245)
TIMELESS rabbit polyclonal (1:1000, Abcam, Cat. No. ab109512)
TIPIN rabbit polyclonal (1:250, Bethyl Laboratories Cat. No. A301-474A)
PARP1 rabbit polyclonal (1:1000, Abcam, Cat. No. ab32138)
PARP2 rabbit polyclonal (1:500, Active Motif, Cat. No 39743)
BRCA2 mouse monoclonal (1:1000, clone 2B, Calbiochem, Cat. No. OP95)
Actinin mouse monoclonal (1:1000, clone AT6/172, Sigma-Aldrich, Cat. No. 05-384)
RNase H1 rabbit polyclonal (1:500, ProteinTech, Cat. No. 15606-1-AP)
FLAG mouse monoclonal (1:1000, clone M2, Sigma Aldrich, Cat. No. F1804)
GFP rabbit polyclonal (1:500, Abcam, Cat. No. ab290).

Secondary Antibodies [Immunofluorescence]:
Alexa Fluor 488 Goat-Anti Rabbit IgG (1:500, Invitrogen, Cat. No., A110334)
Alexa Fluor 488 Goat-Anti Mouse IgG (1:500, Invitrogen, Cat. No. A11001)
Alexa Fluor 594 Goat-Anti Rabbit IgG (1:500, Invitrogen, Cat. No. A11037)
Alexa Fluor 594 Goat-Anti Mouse IgG (1:500, Invitrogen, Cat. No, A11005)
Alexa Fluor 647 Goat-Anti Rabbit IgG (1:500, Invitrogen, Cat. No. A21244)
Alexa Fluor 647 Goat-Anti Mouse IgG (1:500, Invitrogen, Cat. No. A21235)

Secondary Antibodies [Western Blot]:
Anti-Mouse HRP IgG (1:2500, Promega, Cat. No. W401B)
Anti-Rabbit HRP IgG (1:2500, Promega, Cat. No. W402B)

Validation

For antibodies used to monitor DNA damage, we examined cells treated with or without DNA damaging agents. For antibodies used to monitor protein levels by western blot, we validated loss of the protein band in cells transfected with the appropriate siRNA. Specificities of the antibodies were validated by the manufacturer and are listed below:

γH2AX (S139) mouse (Millipore, Cat. No. 05-636): https://www.merckmillipore.com/CH/de/product/Anti-phospho-Histone-H2A.X-Ser139-Antibody-clone-JBW301,MM_NF-05-636
RAD51 rabbit (Bioacademia, Cat. No. 70-002): https://www.bioacademia.co.jp/en/products/list?
53BP1 rabbit (Novus Biologicals, Cat. No. NB100-304): https://www.novusbio.com/products/53bp1-antibody_nb100-304
poly (ADP-ribose) mouse (Trevigen, Cat. No. 4335-MC-100): https://www.rndsystems.com/products/par-padpr-antibody-10ha_4335-mc-100#product-citations
poly (ADP-ribose) mouse (Enzo Life Sciences, Cat. No. ALX-804-220-R100): https://www.enzolifesciences.com/ALX-804-220/poly-adp-ribose-monoclonal-antibody-10h/
PARP1 rabbit (ProteinTech, Cat. No. 13371–1-AP): https://www.ptglab.com/products/PARP1-Antibody-13371-1-AP.htm
PARP2 rabbit (Active Motif, Cat. No. 39743): https://www.activemotif.com/catalog/details/39743/parp-2-antibody-pab
PCNA mouse (Millipore, Cat. No. MABE288): https://www.merckmillipore.com/CH/de/product/Anti-PCNA-Antibody-clone-PC10,MM_NF-MABE288
alpha-Tubulin mouse (Calbiochem, Cat. No. CP06): https://www.merckmillipore.com/CH/de/product/Anti-Tubulin-Mouse-mAb-DM1A,EMD_BIO-CP06
GAPDH mouse (Abcam, Cat. No. ab8245): https://www.abcam.com/products/primary-antibodies/gapdh-antibody-6c5-loading-control-ab8245.html
TIMELESS rabbit (Abcam, Cat. No. ab109512): https://www.abcam.com/products/primary-antibodies/timeless-antibody-epr5275-ab109512.html
TIPIN rabbit (Bethyl Laboratories Cat. No. A301-474A): https://www.thermofisher.com/antibody/product/TIPIN-Antibody-Polyclonal/A301-474A

PARP1 rabbit (Abcam, Cat. No. ab32138): https://www.abcam.com/products/primary-antibodies/parp1-antibody-e102-ab32138.html
BRCA2 mouse (Calbiochem, Cat. No. OP95): https://www.merckmillipore.com/CH/de/product/Anti-BRCA2-Ab-1-Mouse-mAb-2B,EMD_BIO-OP95
Actinin mouse (Sigma-Aldrich, Cat. No. 05-384):https://www.merckmillipore.com/CH/de/product/Anti-Actinin-Antibody-clone-AT6-172,MM_NF-05-384
RNase H1 rabbit (ProteinTech, Cat. No. 15606-1-AP): https://www.ptglab.com/products/RNASEH1-Antibody-15606-1-AP.htm
FLAG mouse (Sigma Aldrich, Cat. No. M2 F1804): https://www.sigmaaldrich.com/CH/de/product/sigma/f1804
GFP rabbit (1:500, Abcam, Cat. No. ab290): https://www.abcam.com/en-at/products/primary-antibodies/gfp-antibody-ab290

Eukaryotic cell lines

Policy information about cell lines and Sex and Gender in Research

Cell line source(s)

HeLa (ATCC, Cat. No.CCL-2)
U2OS (ATCC, Cat. No. HTB-96)
hTERT-RPE1 (ATCC, Cat. No. CRL4000)
DLD1 (ATCC, Cat. No. CCL-221)
DLD1 BRCA2 KO (Horizon, Cat. No. HD 105-007)
PEO1 and PEO4 from Prof. Labidi-Galy (Hospital of the University of Geneva); PEO1 (Sigma-Adrich, Cat. No. 10032308), PEO4 (Sigma-Aldrich, Cat. No. 10032309)
OVSAHO (Sigma-Aldrich, Cat. No. SCC294)
HCT116 (ATCC, Cat. No., CCL-247)
HeLa+RNaseH1-FLAG-DOX from Prof Tarsounas (doi.org/10.1016/j.molcel.2015.12.004)
U2OS T-REx GFP-RNaseH1(D210N)-DOX from Prof Janscak (doi.org/10.1016/j.molcel.2018.11.036)
H1299-shBRCA2-DOX from Prof Tarsounas (https://doi.org/10.1016/j.molcel.2015.12.004)

Authentication

Cell line identity verified by karyotyping.

Mycoplasma contamination

All cell lines regularly tested and found to be negative.

Commonly misidentified lines
(See ICLAC register)

None.

ChIP-seq

Data deposition

☒ Confirm that both raw and final processed data have been deposited in a public database such as GEO.

☒ Confirm that you have deposited or provided access to graph files (e.g. BED files) for the called peaks.

Data access links
May remain private before publication.

GEO Accession Number GSE220223

Files in database submission

EdUseq_Exp5_HeLa_ThymRel_090_siCTRL.fastq.gz, EdUseq_Exp5_HeLa_ThymRel_090_siTIME.fastq.gz, EdUseq_Exp5_HeLa_ThymRel_090_siTIPI.fastq.gz, EdUseq_Exp5_HeLa_ThymRel_120_siCTRL.fastq.gz, EdUseq_Exp5_HeLa_ThymRel_120_siTIME.fastq.gz ,EdUseq_Exp5_HeLa_ThymRel_120_siTIPI.fastq.gz, EdUseq_Exp6_HeLa_ThymRel_120_siCTRL.fastq.gz, EdUseq_Exp6_HeLa_ThymRel_120_siPARP1.fastq.gz, EdUseq_Exp6_HeLa_ThymRel_120_siPARP2.fastq.gz, EdUseq_Exp7_HeLa_ThymRel_120_siCTRL_1.fastq.gz, EdUseq_Exp7_HeLa_ThymRel_120_siCTRL_2.fastq.gz, EdUseq_Exp7_HeLa_ThymRel_120_siPARP1_1.fastq.gz, EdUseq_Exp7_HeLa_ThymRel_120_siPARP1_2.fastq.gz, EdUseq_Exp7_HeLa_ThymRel_120_siPARP2_1.fastq.gz, EdUseq_Exp7_HeLa_ThymRel_120_siPARP2_2.fastq.gz, HeLa_siCTRL_R120_ThyRel_1_nm, HeLa_siCTRL_R120_ThyRel_2_nm, HeLa_siTIME_R120_ThyRel_2_nm, HeLa_siTIPI_R120_ThyRel_2_nm
Processed data of above files have also been submitted as csv and bigwig files

Genome browser session
(e.g. UCSC)

https://www.ncbi.nlm.nih.gov/geo/query/acc.cgi?acc=GSE220223
https://genome.ucsc.edu/

Methodology

Replicates

Control Samples: 7 replicates
TIMELESS depleted samples: 3 replicates
TIPIN depleted samples: 3 replicates
PARP1 depleted samples: 3 replicates
PARP2 depleted samples: 3 replicates

Sequencing depth

#Total reads
EdUseq_Exp5_HeLa_ThymRel_090_siCTRL.fastq.gz: 48200032
EdUseq_Exp5_HeLa_ThymRel_090_siTIME.fastq.gz: 33217880
EdUseq_Exp5_HeLa_ThymRel_090_siTIPI.fastq.gz: 40521243
EdUseq_Exp5_HeLa_ThymRel_120_siCTRL.fastq.gz: 41511519
EdUseq_Exp5_HeLa_ThymRel_120_siTIME.fastq.gz: 36394129
EdUseq_Exp5_HeLa_ThymRel_120_siTIPI.fastq.gz: 38526266
EdUseq_Exp6_HeLa_ThymRel_120_siCTRL.fastq.gz: 24739139

EdUseq_Exp6_HeLa_ThymRel_120_siPARP1.fastq.gz: 20417997
EdUseq_Exp6_HeLa_ThymRel_120_siPARP2.fastq.gz: 21675687
EdUseq_Exp7_HeLa_ThymRel_120_siCTRL_1.fastq.gz: 13091412
EdUseq_Exp7_HeLa_ThymRel_120_siCTRL_2.fastq.gz: 14785436
EdUseq_Exp7_HeLa_ThymRel_120_siPARP1_1.fastq.gz: 14918795
EdUseq_Exp7_HeLa_ThymRel_120_siPARP1_2.fastq.gz: 13095859
EdUseq_Exp7_HeLa_ThymRel_120_siPARP2_1.fastq.gz:14695337
EdUseq_Exp7_HeLa_ThymRel_120_siPARP2_2.fastq.gz: 11962343
HeLa_siCTRL_R120_ThyRel_1_nm: 27940030
HeLa_siCTRL_R120_ThyRel_2_nm: 25907850
HeLa_siTIME_R120_ThyRel_2_nm: 24287805
HeLa_siTIPI_R120_ThyRel_2_nm: 22040707

#Uniquely mapped reads
EdUseq_Exp5_HeLa_ThymRel_090_siCTRL.fastq.gz: 34067038
EdUseq_Exp5_HeLa_ThymRel_090_siTIME.fastq.gz: 25563815
EdUseq_Exp5_HeLa_ThymRel_090_siTIPI.fastq.gz: 30140809
EdUseq_Exp5_HeLa_ThymRel_120_siCTRL.fastq.gz: 31173814
EdUseq_Exp5_HeLa_ThymRel_120_siTIME.fastq.gz: 27136579
EdUseq_Exp5_HeLa_ThymRel_120_siTIPI.fastq.gz: 28916857
EdUseq_Exp6_HeLa_ThymRel_120_siCTRL.fastq.gz: 13159181
EdUseq_Exp6_HeLa_ThymRel_120_siPARP1.fastq.gz: 12501152
EdUseq_Exp6_HeLa_ThymRel_120_siPARP2.fastq.gz: 11817676
EdUseq_Exp7_HeLa_ThymRel_120_siCTRL_1.fastq.gz: 7058178
EdUseq_Exp7_HeLa_ThymRel_120_siCTRL_2.fastq.gz: 8226376
EdUseq_Exp7_HeLa_ThymRel_120_siPARP1_1.fastq.gz: 8116157
EdUseq_Exp7_HeLa_ThymRel_120_siPARP1_2.fastq.gz: 7276690
EdUseq_Exp7_HeLa_ThymRel_120_siPARP2_1.fastq.gz: 7968444
EdUseq_Exp7_HeLa_ThymRel_120_siPARP2_2.fastq.gz: 6690834
HeLa_siCTRL_R120_ThyRel_1_nm: 21631573
HeLa_siCTRL_R120_ThyRel_2_nm: 21266081
HeLa_siTIME_R120_ThyRel_2_nm: 19973301
HeLa_siTIPI_R120_ThyRel_2_nm: 18200703

Read length: 100 bp
Single-end reads.

Antibodies

We did not use antibodies. Nascent DNA was labeled with EdU and the EdU-labeled DNA was then linked to biotin using Click-iT Chemistry.

Peak calling parameters

We did not call peaks. We used gene annotation data (refseq from NCBI) to align the transcription start sites of large genes and then monitor their replication in early S phase.

Data quality

We did not call peaks.

Software

The data were visualized using custom scripts that have been submitted in the supplementary data section of Macheret and Halazonetis, Nature 2018

Flow Cytometry

Plots

Confirm that:

☒ The axis labels state the marker and fluorochrome used (e.g. CD4-FITC).

☒ The axis scales are clearly visible. Include numbers along axes only for bottom left plot of group (a 'group' is an analysis of identical markers).

☒ All plots are contour plots with outliers or pseudocolor plots.

☒ A numerical value for number of cells or percentage (with statistics) is provided.

Methodology

Sample preparation

Cells were harvested by trypsinization and fixed in 90% methanol overnight at -20° C. EdU detection was performed using the Click-it EdU Alexa Fluor 647 Flow Cytometry Assay Kit (Invitrogen Cat. No. C-10424) according to the manufacturer's instructions. Detection of γH2AX phosphorylation was performed using the Guava Histone H2AX Phosphorylation Assay Kit (Luminex, FCCS100182) according to the manufacturer's instructions. The genomic DNA was stained by incubating the cells in PBS containing RNase (Roche, Cat. No. 11119915001) and propidium iodide (PI) (Sigma-Aldrich Cat. No. 81845).

Instrument

Gallios, Model 2L/8C, Beckman Coulter

Software

Kaluza, version 2.1, Beckman Coulter

Cell population abundance | At least 20,000 cells were evaluated per sample.

Gating strategy | Cells were gated by FSC/SSC, then by PI peak area height/PI peak height to eliminate clumped cells, then by EdU and by gH2AX signals. An example showing one sample is attached. This example is provided as Supplementary Figure 2.

☒ Tick this box to confirm that a figure exemplifying the gating strategy is provided in the Supplementary Information.
